# Establishment of a rat model for uterine leiomyomas based on Western and traditional Chinese medicine theories

**DOI:** 10.1590/1414-431X20187627

**Published:** 2018-07-02

**Authors:** Hui Zhao, Yao Li, QiuXia Xu, Fu Peng, JinShuang Zhao, R. Clinton Webb, Cheng Peng, ChengHao Yu

**Affiliations:** 1 Chengdu University of Traditional Chinese Medicine, Chengdu, China; 2 Department of Physiology, Medical College of Georgia, Augusta University, Augusta, GA, USA

**Keywords:** Uterine leiomyomas, Animal model, Traditional Chinese medicine

## Abstract

Uterine leiomyomas (ULs) are benign monoclonal tumors that arise from the underlying myometrial tissue in the uterus. Effective therapies are still lacking because of poor understanding of the pathophysiology and epidemiology. Hence, it is urgent to establish efficient animal models to screen novel anti-UL therapies. In this study, for the first time, traditional Chinese medicine and Western medicine were combined to establish an animal model of ULs in rats. In order to evaluate the function and value of the novel model, it was compared with other models. The long-term and short-term rat models for ULs were established using progesterone and diethylstilbestrol. Rats in Qi stagnation and blood stasis group were injected with epinephrine hydrochloride and received chronic unpredictable stress for two weeks. Rats in combining disease with syndrome group (CDWSG) received not only epinephrine hydrochloride injection and chronic unpredictable stress but also progesterone and diethylstilbestrol treatment. We analyzed differences in organ coefficient, uterus size, uterine pathology, concentrations of progesterone, estradiol, progesterone receptor, estrogen receptor, expression of desmin, α-smooth muscle actin, and vimentin among the five groups. The animal model of ULs was successfully constructed by loading the rats with estrogen and progesterone. The rat model of CDWSG was more stable than other groups and the method was the most efficient.

## Introduction

Uterine leiomyomas (ULs) are benign monoclonal tumors that arise from the underlying myometrial tissue in the uterus (1). Although the incidence rate is about 25% among women of reproductive age (2), the cellular and molecular mechanism(s) by which ULs develop are not fully understood. The common treatment for ULs, hysterectomy, is not an acceptable option for many women who want to preserve their fertility (3). Thus, it is urgent to establish efficient animal models to screen novel anti-UL therapies, which include new drugs and potential non-hormonal therapeutics for medical treatment. Currently, accumulating evidence indicates that gonadotropins (4), adipokines (5), and ovarian peptides (6) are the key factors involved in leiomyoma onset and growth. Likewise, abundant preclinical and clinical reports indicate that the levels of estradiol (E_2_) and progesterone (P) could serve as major promoters of leiomyoma development and growth (7).

ULs belong to the concept of “Zhengjia” and “Jiju”, which mean that they are caused by “blood stasis” and “Qi stagnation” in traditional Chinese medicine (TCM). In TCM culture, Qi is an active principle forming part of any living creature and the blood circulation relies on the promotion of Qi. The slowing or pooling of blood because of Qi stagnation is identified as “blood stasis”. According to the TCM theory, Qi stagnation and blood stasis in the uterus can lead to ULs. Chen (8) found that “blood stasis” and “Qi stagnation” are the most common syndromes in UL patients. The results indicated that the most efficient therapeutic method was to promote blood circulation and remove blood stasis.

Most mechanisms that have been proposed for the development of ULs in patients are based on animal studies, which present a powerful tool to help investigators develop models for disease mechanism. This study aimed to establish, for the first time, a UL model in rats using the TCM theory of UL pathogenesis.

## Material and Methods

### Experimental animals

Non-pregnant female Sprague-Dawley rats with an average body weight of 200 g were provided by Chengdu Dashuo Experimental Animal Tech (China). The experimental animal licensing SCXK (chuan) 2013–24 and SYXK (chuan) 2014–049 were used. Rats had adaptive feeding with normal light conditions and suitable humidity and temperature for 3 days. All of the animal protocols were performed in strict accordance with the guidelines for the care and use of laboratory animals established by the Animal Ethical Committee of Chengdu University of Traditional Chinese Medicine. During the experiments, efforts were made to minimize both animal suffering and the number of animals used.

### Drugs and reagents

Diethylstilbestrol was obtained from Chengdu West Chemical Industry (China). Progesterone was purchased from Zhejiang Xian-Ju Pharmaceuticals (China). Absolute ethyl alcohol, xylene, aluminum potassium sulfate, sodium iodate, hydrochloric acid, potassium dichromate, and concentrated sulfuric acid were obtained from Chengdu Ke Long Chemical Industry (China). Paraformaldehyde was purchased from China National Group Corporation (China). PBS (0.01 M, pH 7.2–7.4), desmin, rabbit polyclonal antibody (1:200), α-SMA, rabbit polyclonal antibody (1:100), vimentin, goat anti-rabbit IgG, biotin (1:100), DAB kit, and citrate buffer solution (0.01 M, pH 6.0) were obtained from Beijing Zhong Shan Golden Bridge Biotechnology (China). Hematoxylin was obtained from Beijing J&K Scientific (China). Glycerin was purchased from Tianjin Fu Yu Fine Chemical Industry (China). Eosin was obtained from Tokyo Chemical Industry Co., Ltd (Japan) and neutral balsam from Shanghai Yi Yang Instruments (China).

### Induction of a rat model of ULs

The rats were randomly divided into five groups: normal control group (NCG), long-term model group (LTMG), short-term model group (STMG), combining disease with syndrome group (CDWSG), and Qi stagnation and blood stasis group (QSABSG). Rats in the NCG were given distilled water by gavage once a day (2 mL/day) and injected with normal saline (0.05 mL/day) through the lower limb lateral muscle 3 times a week for 5 weeks. Rats in the LTMG were given diethylstilbestrol (0.167 mg/kg) by gavage on Monday, Wednesday, and Friday, and injected with 1.0 mg of progesterone through the lower limb lateral muscle every Sunday for 20 weeks. In the STMG, rats were given diethylstilbestrol (1.35 mg·kg^-1^·d^-1^) and injected with 1.0 mg of progesterone through the lower limb lateral muscle on Monday, Wednesday, and Friday for 5 weeks. Rats in the CDWSG received diethylstilbestrol (1.35 mg/kg) every day, and were injected with 1.0 mg progesterone in the lateral leg on Monday, Wednesday, and Friday for 5 weeks. Then, rats were injected with 0.9 mg·kg^-1^·d^-1^ adrenal hydrochloride from the fourth week, and given external stimulation four hours later every day (1: 60 dB noise for 3 h; 2: Day reversal; 3: Swimming in 5–10°C water for 4 min; 4: Hanging from tail for 10 min; 5: Heat exposure for 10 min at 50°C) every day. Each stimulus was performed at least 2 times throughout the cycle of two weeks. The rats in QSABSG received a subcutaneous injection of epinephrine hydrochloride (0.9 mg·kg^-1^·d^-1^) and were given stimulation as for CDWSG.

### Tissue and sample preparation

The rats were kept fasted for 12 h after the last administration; water was available. Blood samples were withdrawn from the arteria cruralis and stored at 4°C for 1h. Then, blood samples were centrifuged at 5–6°C for 10 min (1500 *g*), and the supernatant was stored at -80°C. After the rats were sacrificed, the uterus and ovary were weighed and measured after dissection. Uteruses were divided into two then stored in liquid nitrogen and fixative. The diameter of the thickest point above the cervix and the length between ovary and cervix were measured (mm) with Vernier calipers.

### Calculation of the uterine coefficient

The organ coefficient was calculated using the following formula: organ coefficient = organ weight (mg) / rat body weight (g) × 100.

### Testing the pathological tissue of uterus

The most intumescent part on the uterus was obtained, fixed in 4% formaldehyde, dehydrated, embedded in paraffin, and sectioned. Sliced tissues were processed as follows: dewaxing and hydration; staining with hematoxylin for 10–20 min and rinsing for 1–3 min; differentiating with 1% hydrochloric acid for 5–10 s and rinsing for 1–3 min; putting tissues in phosphate-buffered saline to return to blue and rinsing for 1–3 min; putting tissues in 85% ethylene alcohol for 3–5 min; staining with eosin for 3–5 min and rinsing for 3–5 s; dehydrating with 80, 90, 95, and 100% graded alcohol; vitrification by dimethylbenzene; and sealing with neutral balsam.

### Enzyme-linked immunosorbent assay (ELISA)

ELISA was used to detect E_2_, P, estrogen receptor (ER), and progesterone receptor progesterone receptor (PR) in serum, uterus homogenate, and ovary homogenate. Serum was taken from the refrigerator, and diluted with diluent after reaching room temperature. The sample solution was introduced into the well, mixed, and stained for 1 h (37°C). Scrubbing solution was diluted and put into the well after the sample solution was taken out. After washing, color-developing agent and terminating agent were added to the well. Absorbance was detected using 450 nm wave length. (Amyjet Scientific Inc., China)

### Immunohistochemistry

Immunohistochemistry was used to detect the expression of desmin, vimentin, and α-smooth muscle actin. Trinocular photomicroscope (BA400 Digital Motic China Group Co., Ltd., China) was used to collect pictures.

### Statistical analyses

Data are reported as means±SD and were analyzed with one-way ANOVA (IBM SPSS 21.0, USA). Results were considered statistically significant if the P value was lower than 0.05.

## Results

### Macroscopic changes of uterus

**Figure 1.**
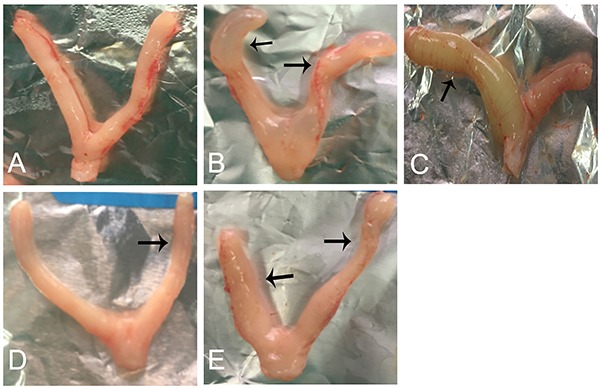
Macroscopic changes of uterus. The uteruses in NCG (*A*) and QSABSG (D) had uniform texture, bright color, symmetrical Y shape and did not show cysts, tubercle or tumefaction. Many uteruses in STMG (B), LTMG (*C*), and CDWSG (*E*) had cysts, nodules, and swelling (arrows). NCG: normal control group; STMG: short-term model group; LTMG: long-term model group; QSABSG: Qi stagnation and blood stasis group; CDWSG: combining disease with syndrome group.

Uteruses in NCG (Figure 1A) had regular texture, bright color with no cysts, nodules, or swelling. However, the uteruses in CDWSG (Figure 1E), STMG (Figure 1B), and LTMG (Figure 1C) were asymmetric, had faded color and obvious cysts, nodules, and swelling. In QSABSG (Figure 1D), uteruses had no nodules and swelling but had faded color and asymmetry in length. The model approach in CDWSG, STMG, and LTMG caused cysts, nodules, and swelling of the rat uterus.

### Pathologic changes in UL tissue

**Figure 2.**
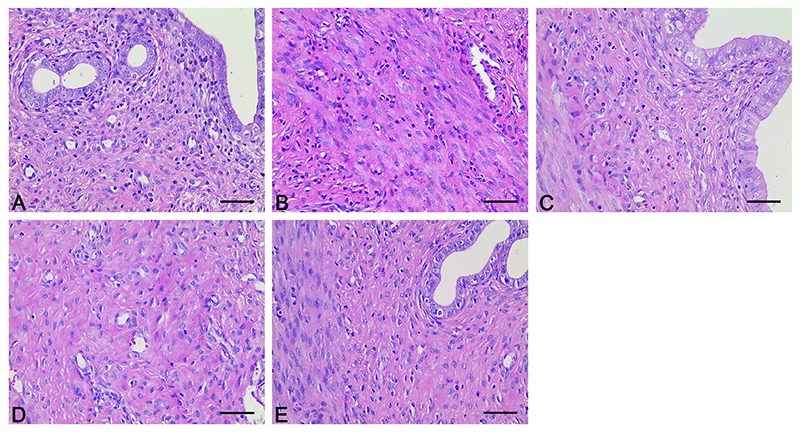
Pathologic changes in uterine leiomyomas tissue. Endometrium in NCG (*A*) and QSABSG (*D*) were intact with no hyperplasia, atrophy, edema, denaturation or necrosis. In CDWSG (*E*) and LTMG (*C*), the endometrial epithelial cells had vacuolar degeneration, the smooth muscle cells had focal proliferation and hyaline degeneration, and the muscle was infiltrated by inflammatory cells. In STMG (*B*), uterine smooth muscle cells had little hyaline degeneration, and muscle layer had inflammatory cells. NCG: normal control group; STMG: short-term model group; LTMG: long-term model group; QSABSG: Qi stagnation and blood stasis group; CDWSG: combining disease with syndrome group. Magnification bar: 50 μm.

The endometrium in NCG (Figure 2A) and QSABSG (Figure 2D) was intact with no hyperplasia, atrophy, edema, denaturation, or necrosis. In CDWSG (Figure 2E) and LTMG (Figure 2C), the endometrial epithelial cells presented vacuolar degeneration, the smooth muscle cells had focal proliferation and hyaline degeneration, disordered arrangement, and unequal thickness. The muscle was infiltrated by inflammatory cells. The mucosa in LTMG showed eosinophilic infiltration, and the mucosal epithelial cells had slightly increased proliferation (Figure 2C). In STMG, uterine smooth muscle cells had little hyaline degeneration and disordered arrangement, and the muscle layer presented inflammatory cells with unequal thickness (Figure 2B). The model approach in CDWSG, STMG, and LTMG caused changes of uterine histology such as degeneration and proliferation.

### Uterus and ovary organ coefficients

**Figure 3.**
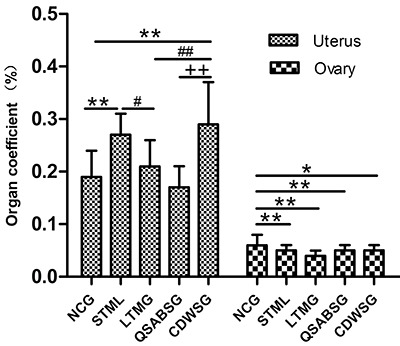
Effect of treatment on organ coefficients of the uterus and ovary. Data are reported as means±SD. *P<0.05, **P<0.01 compared with NCG; ^#^P<0.05, ^##^P<0.01 compared with LTMG; ^++^P<0.01 compared with QSABSG (ANOVA). NCG: normal control group; STMG: short-term model group; LTMG: long-term model group; QSABSG: Qi stagnation and blood stasis group; CDWSG: combining disease with syndrome group.

The uterine organ coefficient in CDWSG increased compared with NCG, LTMG, and QSABSG (P<0.01). The uterus coefficient in STMG was higher than that in NCG (P<0.01) and in LTMG (P<0.05). The uterus coefficient did not differ significantly between NCG and LTMG (P>0.05). Compared with NCG, ovary organ coefficient of the other groups decreased significantly (Figure 3). The reason for uterine organ coefficient increase might be hyperplasia and edema. Ovary organ coefficient decreased after treatment, which caused injury to the ovary.

### Transverse diameter (r) and diameter (R) of uterus

**Figure 4.**
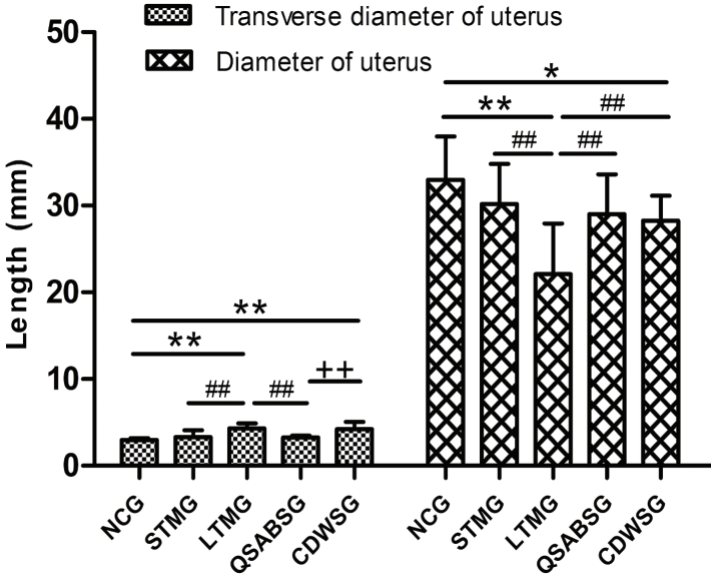
Changes of transverse diameter and diameter of uterus after modeling. Data are reported as means±SD. *P<0.05, **P<0.01 compared with NCG; ^##^P<0.01 compared with LTMG; ^++^P<0.01 compared with QSABSG (ANOVA). NCG: normal control group; STMG: short-term model group; LTMG: long-term model group; QSABSG: Qi stagnation and blood stasis group; CDWSG: combining disease with syndrome group.

Compared with NCG, uterine transverse diameter in LTMG and CDWSG increased (P<0.01), and the difference between LTMG and CDWSG was not significant. Uterine transverse diameter in LTMG was greater than that in STMG and QSABSG (P<0.01). In CDWSG, it was greater than that in QSABSG (P<0.01), and the difference between STMG and CDWSG was not significant (P>0.05). The measurement did not differ significantly among NCG, STMG, and QSABSG (P>0.05). Compared with NCG, uterine diameter in LTMG (P<0.01) and CDWSG (P<0.05) decreased. Uterine diameter did not differ significantly among NCG, STMG, and QSABSG (P>0.05), and in LTMG it was lower than in STMG, CDWSG, and QSABSG (P<0.01) (Figure 4).

### E_2_ and P concentrations in serum

**Figure 5.**
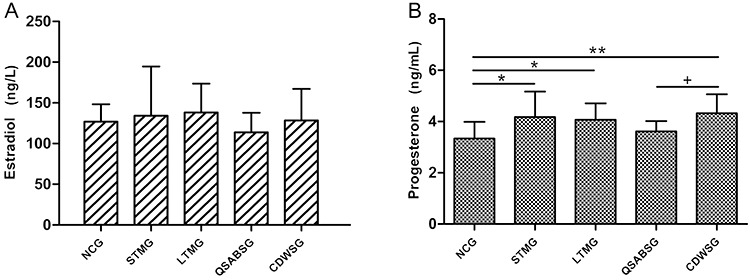
Effect of treatment on estradiol and progesterone concentration in serum. Data are reported as means±SD. *P<0.05, **P<0.01 compared with NCG; ^+^P<0.05 compared with QSABSG (ANOVA). NCG: normal control group; STMG: short-term model group; LTMG: long-term model group; QSABSG: Qi stagnation and blood stasis group; CDWSG: combining disease with syndrome group.

E_2_ concentration in all groups did not differ significantly (P>0.05) (Figure 5A). Compared with NCG, P concentration increased in STMG (P<0.05), LTMG (P<0.05), and CDWSG (P<0.01). P concentration in STMG, LTMG, and CDWSG did not differ significantly (P>0.05). P concentration in CDWSG was higher than in QSABSG (Figure 5B).

### E_2_, P, ER, and PR concentration in the uterus and ovary

**Figure 6.**
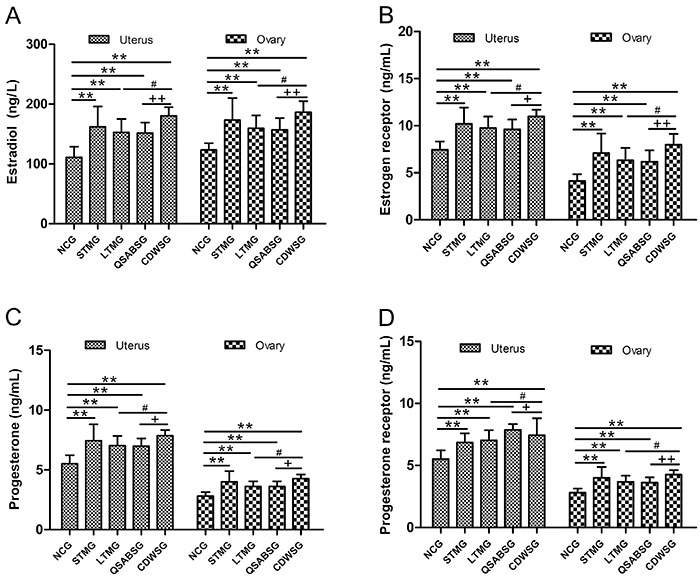
Effect on estradiol, progesterone, estrogen receptor, and progesterone receptor concentration in the uterus and ovary. Data are reported as means±SD. **P<0.01 compared with NCG; ^#^P<0.05 compared with LTMG; ^+^P<0.05, ^++^P<0.01 compared with QSABSG (ANOVA). NCG: normal control group; STMG: short-term model group; LTMG: long-term model group; QSABSG: Qi stagnation and blood stasis group; CDWSG: combining disease with syndrome group.

Compared with the control group, E_2_, P, ER, and PR in the uterus and ovary of CDWSG, LTMG, STMG, and QSABSG increased (P<0.01) and did not differ significantly among LTMG, STMG, and QSABSG (P>0.05). P, ER, and PR concentrations in the uterus of CDWSG were higher than those in LTMG and QSABSG (P<0.05). E_2_ concentration in the uterus of CDWSG was higher than in LTMG (P<0.05) and QSABSG (P<0.01). E_2_, ER, and PR concentrations in the ovary of CDWSG were higher than in LTMG (P<0.05) and QSABSG (P<0.01). P concentration in the ovary of CDWSG was higher than that in LTMG and QSABSG (P<0.05) (Figure 6A-D).

### Immunohistochemistry

**Figure 7.**
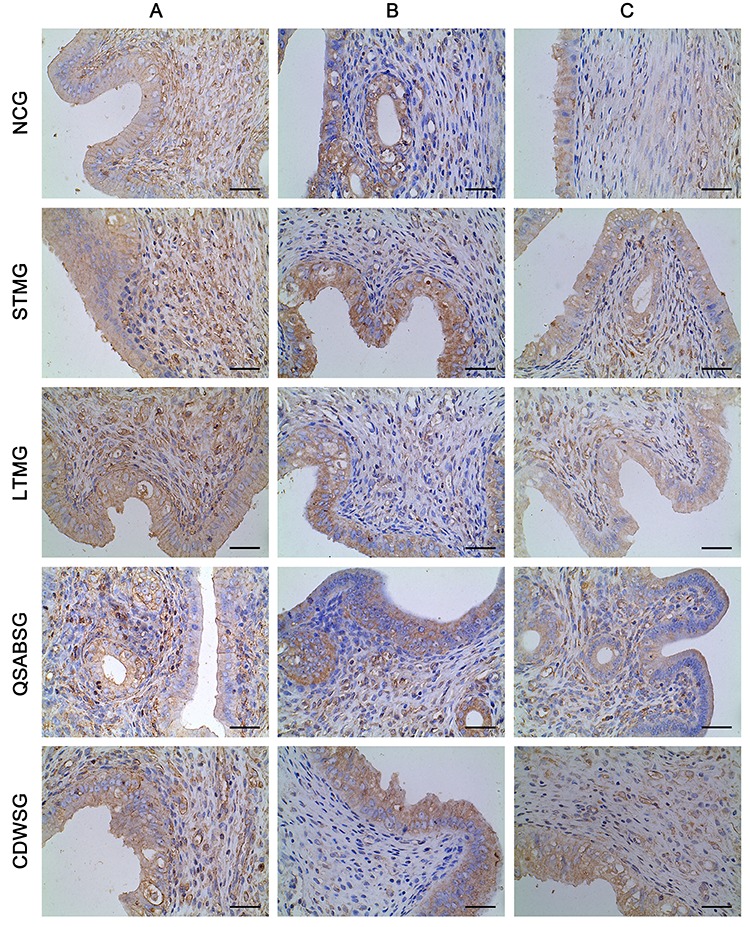
Immunohistochemical characteristics. The tumor cells in STMG, LTMG, and CDWSG were positive for α-SMA (*A*), desmin (*B*), and vimentin (*C*). NCG: normal control group; STMG: short-term model group; LTMG: long-term model group; QSABSG: Qi stagnation and blood stasis group; CDWSG: combining disease with syndrome group. Magnification bars: 40 μm.

Analysis showed strong immunoreactivity for α-smooth muscle actin (Figure 7A), desmin (Figure 7B), and vimentin (Figure 7C).

## Discussion

ULs are benign smooth muscle tumors of the uterus, which have a lifetime incidence of approximately 70% in the general population (9). At present, abundant evidence acknowledges E_2_ and P as ultimate elements involved in the formation of leiomyomas. Matsuo et al. (10) demonstrated that estradiol and progesterone promoted proliferation of leiomyoma cells *in vitro*. Ono et al. (11) found that E_2_ and P stimulated complex paracrine signals that allowed leiomyoma cells to release mitogenic signals to the adjacent immature cells. When leiomyoma tissues from patients were transplanted into ovariectomized, immunodeficient mice, treatment with the combination of E_2_ and P would increase tumor size (12).

TCM believes that “blood stasis” and “Qi stagnation”, which are due to upsetting emotions, are common syndromes. TCM considers that anger and worry always cause stagnation of liver Qi and results in blood stasis among women. For example, extreme physical weakness and long-term worries may cause Qi stagnation (13), further induce blood stasis, and lead to mass growth (zhengjia). Epinephrine hydrochloride has been used frequently to induce a “blood stasis” model. Rats that received subcutaneous injection of epinephrine hydrochloride and cold water stimulation presented changes of hemorheology and damage of vascular endothelium (14–17). Rats in CDWSG and QSABSG received subcutaneous injection of epinephrine hydrochloride and unpredicted stimulation to try to copy the uterine leiomyoma model. Unpredicted stimulation was achieved by changing external environmental factors such as light, noise, and temperature to enrage rats to imitate depressing and anger states of humans.

In STMG, LTMG, and CDWSG, concentrations of P in serum and of P, E2, PR, ER in the uterus and ovary increased, which is similar to pathological features of human uterine leiomyomas (18,19). Uterine transverse diameter in these three groups increased significantly and uterine diameter reduced significantly. Animal models of uterine leiomyoma in STMG, LTMG, and CDWSG were constructed successfully. Although UL rat models were established with shorter time in CDWSG, uterus coefficient and concentration of E2, P, ER, and PR in the uterus and ovary in CDWSG were significantly increased compared with LTMG. The rats with a long-time interference of estrogen and progesterone demonstrated higher mortality, indicating that the long-time intervention would be harmful to the health and survival of model animals. In CDWSG and STMG, uterus coefficient, concentration of E_2_ and P in serum, concentration of E2, P, ER, and PR in the uterus and ovary had a tendency to increase, although not significantly.

ULs were not induced in QSABSG, as the uteruses did not present cysts, tubercle or tumefaction. However, concentration of P and E2 in serum and concentration of P, E2, PR, and ER in the uterus and ovary increased. Liao et al. (20) suggested that abnormal change of emotions was a risk factor for uterine fibroids, and our study can provide important evidence for this viewpoint. In addition, epinephrine caused a holistic influence rather than a specific effect that might lead to disease.

In conclusion, firstly, there are many methods for inducing animal uterine leiomyoma such as spontaneous model, transgenic model, xenograft model, estrogen and progesterone stimulation model, and surgically induced model (21,22 [Bibr B23] [Bibr B24] [Bibr B25]). However, few studies established an animal model of ULs based on TCM theory. Therefore, the combination of sex hormone stimulation with external stimulation was a great innovation related to TCM theory. Secondly, according to our study, animal models of ULs were constructed successfully by combining estrogen and progesterone loading with TCM theory. Furthermore, the establishment of the animal model of ULs in CDWSG was more obvious and efficient. Therefore, we recommend researchers to adopt the combination of disease with syndrome for further pharmacodynamic and mechanism studies about ULs.
